# Gantry-period-guided modulation factor selection in helical tomotherapy: effects on delivery efficiency, dosimetry and verification accuracy

**DOI:** 10.3389/fonc.2026.1891272

**Published:** 2026-08-14

**Authors:** Xiaoli Jin, Tianhui Gao, Xiangqian Liu, Hailiang Zhang, Xin Yang, Xia Zhang

**Affiliations:** 1 Department of Oncology, Shanxi Bethune Hospital, Shanxi Academy of Medical Sciences, Third Hospital of Shanxi Medical University, Tongji Shanxi Hospital, Taiyuan, China; 2 Department of Biomedical Engineering, Shanxi Medical University, Taiyuan, China; 3 Department of Equipment Management and Maintenance Center, Shanxi Bethune Hospital, Shanxi Academy of Medical Sciences, Third Hospital of Shanxi Medical University, Tongji Shanxi Hospital, Taiyuan, China

**Keywords:** gantry period, helical tomotherapy, leaf open time, modulation factor, patient-specific quality assurance, treatment efficiency

## Abstract

**Purpose:**

To explore a gantry-period-guided strategy for identifying a lower useful modulation factor (MF) in helical tomotherapy under fixed field width and pitch.

**Methods:**

Twenty previously treated cases from head, head-and-neck, abdomen and pelvis were replanned with a field width of 2.5 cm and a pitch of 0.287. MF was initialized at 5.0 and reduced by 0.2 with unchanged objectives. At each level, plans were reoptimized and clinically reviewed. MF_limit_ was defined as the last clinically acceptable MF before the step-down sequence entered the mechanical gantry-period plateau (~11.8 s); MF_min_ was the lowest clinically acceptable MF identified after further reduction. Gantry period, delivery time, leaf-open-time (LOT) metrics, dosimetric indices and pretreatment gamma pass rates were compared.

**Results:**

All cases entered the efficiency-limited pathway. MF_limit_ ranged from 1.9 to 2.8. MF reduction produced a biphasic gantry-period response, with an initial decrease followed by a mechanical plateau. Further reduction from MF_limit_ to MF_min_ did not meaningfully reduce delivery time. LOT_max_ decreased and LOT_mean_ remained relatively stable, whereas T_gap_, defined as the temporal gap between the available projection time and LOT_max_, increased markedly at MF_min_, indicating unused projection time after plateau entry. Compared with MF_min_, MF_limit_ was associated with improved PTV homogeneity and conformity, more favorable exploratory pooled OAR dose trends, and higher 1%/1 mm gamma pass rates, while 3%/2 mm and 2%/2 mm pass rates were similar.

**Conclusions:**

The MF immediately before gantry-period plateau entry may provide a practical reference point for limiting MF reduction. In this pilot cohort, this gantry-period-guided approach helped maintain plan quality and avoid reductions in modulation that offered little efficiency benefit. Further studies across additional planning configurations and disease sites will be needed to confirm its broader applicability.

## 1 Introduction

Helical tomotherapy (HT) is an established modality for intensity-modulated radiotherapy, characterized by continuous gantry rotation, couch translation, and binary multileaf collimator modulation. During delivery, radiation fluence is modulated through leaf open times (LOTs) across multiple projections, enabling highly conformal target coverage and organ-at-risk (OAR) sparing (1).

In HT treatment planning, plan quality and delivery efficiency are strongly influenced by machine-related parameters, mainly field width (FW), pitch, and modulation factor (MF) (2). FW is a major determinant of treatment time (3), whereas pitch affects longitudinal dose continuity and the risk of thread effects (4, 5). MF, defined as the ratio of the maximum LOT to the mean LOT, controls the degree of fluence modulation allowed during optimization (6). A higher MF may increase modulation flexibility and improve dose shaping, but it can also prolong delivery. Conversely, an excessively low MF may restrict modulation and compromise dosimetric quality.

A practical challenge in routine HT planning is to determine how far MF can be reduced without compromising plan quality. Once the theoretical gantry period reaches the mechanical lower limit of the treatment unit, further MF reduction is unlikely to improve delivery efficiency. At this point, MF reduction may still alter the internal LOT distribution, but it no longer shortens the treatment time. Therefore, the gantry-period plateau may provide a clinically meaningful reference for identifying the lower useful bound of MF.

Several MF selection approaches have been proposed (7–13). Previous studies have used LOT distribution statistics (11), institutional averages of actual MF (12), or Pareto-front analyses based on multiple combinations of pitch and MF (13). These approaches have provided valuable insight into MF optimization; however, some are planning-intensive or difficult to implement for every patient in daily clinical practice. In many institutions, MF selection remains empirical and is often based on treatment site or planner experience.

The aim of this study was to explore a simple gantry-period-guided MF selection strategy for HT under fixed FW and pitch. For each case, MF was progressively reduced until the step-down sequence approached the first limiting condition: either further reduction led to entry into the mechanical gantry-period plateau while plan quality remained clinically acceptable, or clinical acceptability was lost before plateau entry. For efficiency-limited cases, the last clinically acceptable MF before plateau entry was defined as MF_limit_ and evaluated as a patient-specific lower practical limit for MF selection. Rather than identifying a globally optimal MF through extensive multi-parameter planning, this study focused on determining the point at which additional MF reduction is unlikely to provide further efficiency gains.

## 2 Materials and methods

### 2.1 Dataset and planning settings

Twenty previously treated cases were retrospectively included in this in-silico planning study. The cohort comprised four anatomical regions: head, head and neck, abdomen, and pelvis, with five cases in each group. Prescription doses ranged from 1.7 to 2.6 Gy per fraction. Patient characteristics and prescription information are summarized in **Table 1**.

**Table 1:** Clinical characteristics and prescription information for the 20 evaluated cases.

Case	Site	Prescription dose	Description
1	Head	57.2/39.6 Gy in 22 fractions	Whole brain + local boost (no brainstem involvement)
2	Head	60/54 Gy in 30 fractions	Brain metastases (PTV overlapping with brainstem)
3	Head	60 Gy in 30 fractions	Nasal cavity NKT lymphoma
4	Head	54 Gy in 27 fractions	Pituitary tumor
5	Head	60/54 Gy in 30 fractions	Glioma
6	Head & Neck	60/54 Gy in 30 fractions	Laryngeal cancer
7	Head & Neck	69.96/60.06 Gy in 33 fractions	Nasopharyngeal carcinoma
8	Head & Neck	69.96/59.4 Gy in 33 fractions	Mandibular cancer
9	Head & Neck	66/54 Gy in 30 fractions	Nasal cavity tumor
10	Head & Neck	66/60/54 Gy in 30 fractions	Laryngeal cancer
11	Abdomen	50 Gy in 25 fractions	Liver cancer
12	Abdomen	60/54 Gy in 30 fractions	Gastric cancer
13	Abdomen	56 Gy in 28 fractions	Pancreatic carcinoma
14	Abdomen	50.4 Gy in 28 fractions	Retroperitoneal lymph node metastasis
15	Abdomen	50 Gy in 25 fractions	pancreatic carcinoma
16	Pelvis	60/45 Gy in 25 fractions	Cervical cancer
17	Pelvis	50 Gy in 25 fractions	Cervical cancer
18	Pelvis	61.6/50.4 Gy in 28 fractions	Cervical cancer
19	Pelvis	60/50 Gy in 25 fractions	Cervical cancer + retroperitoneal lymph node metastasis
20	Pelvis	50 Gy in 25 fractions	Rectal cancer

For each case, experimental plans were generated using the original CT images, structure sets, optimization objectives, and dose constraints from the corresponding clinical plan. All plans were created in Accuray Planning Station version 5.1.8.23 using the GPU-based VoLO optimizer. To isolate the effect of MF, the field width and pitch were fixed at 2.5 cm and 0.287, respectively, for all experimental plans. The original clinical plans used the same field width and pitch settings.

### 2.2 Identification of MF_limit_

**Figure 1:**
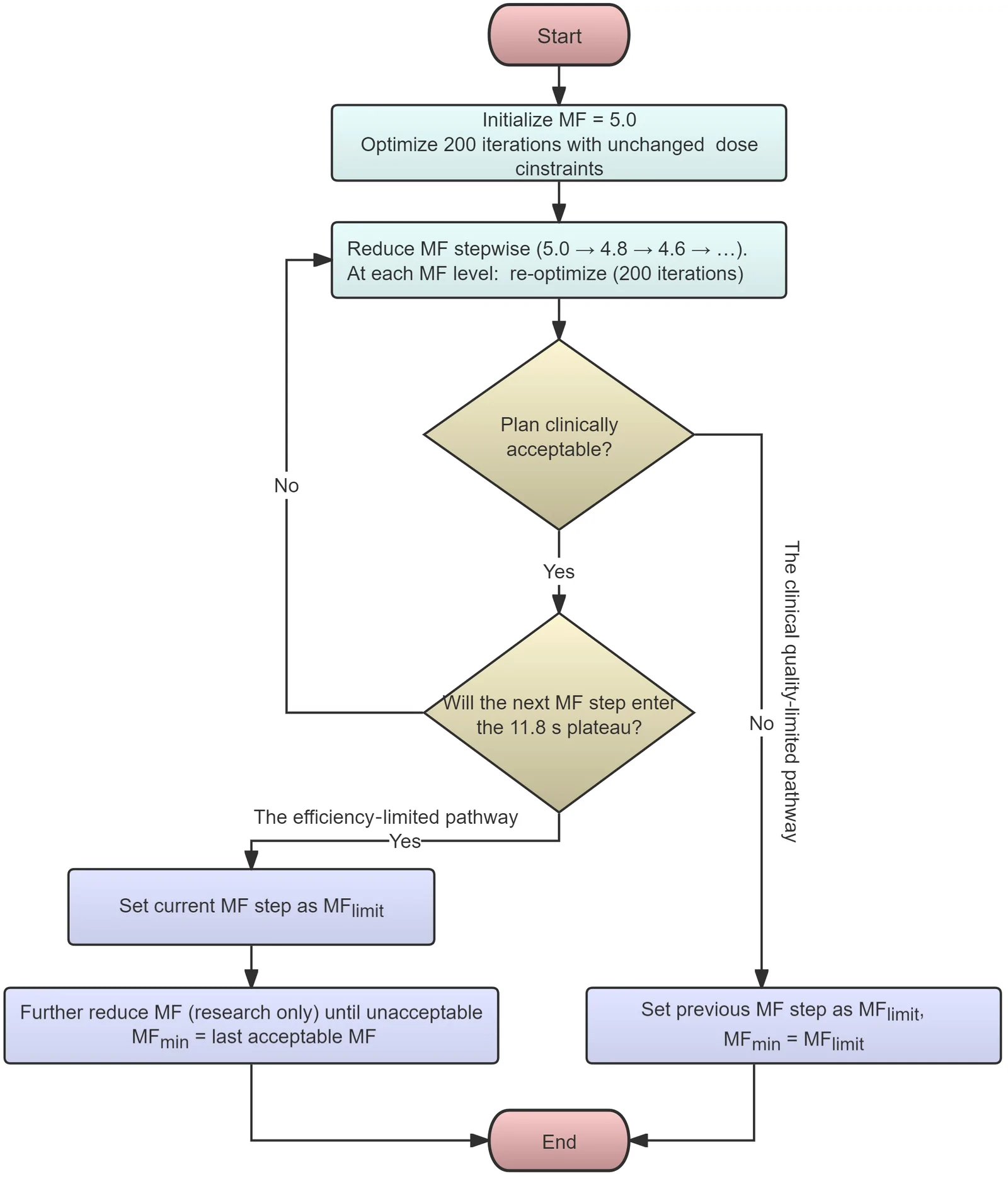
Workflow for identifying MF_limit_ using the gantry-period-guided step-down strategy. The modulation factor (MF) was initialized at 5.0 and reduced in steps of 0.2. At each MF level, the plan was reoptimized for 200 iterations with unchanged dose constraints and evaluated for clinical acceptability. If a plan became clinically unacceptable before reaching the gantry-period plateau, the previous MF step was designated as MF_limit_, and MF_min_ was identical to MF_limit_ (clinical quality-limited pathway). If the plan remained acceptable, the next MF step was examined to determine whether it would enter the mechanical gantry period plateau. When plateau entry was predicted, the current MF was designated as MF_limit_ (efficiency-limited pathway). For research purposes, MF reduction continued beyond MF_limit_ to determine MF_min_, defined as the lowest MF value producing a clinically acceptable plan.

A step-down workflow was used to identify the patient-specific lower practical limit of MF, denoted MF_limit_. The workflow is illustrated in **Figure 1**.

First, the MF was set to 5.0, the maximum value allowed by the planning system. This plan was re-optimized for 200 iterations using unchanged optimization objectives and dose constraints, followed by high-resolution dose calculation. The resulting plan was labeled P-MF_5_. To ensure that all plans were fully converged prior to comparison, a uniform setting of 200 optimization iterations was applied for every MF level. This decision was guided by the planning workflow described by De Kerf et al. (13). To confirm that this iteration number was also sufficient under the planning environment of the present study, one representative case from each anatomical region was reoptimized with 25, 50, 150, and 200 iterations. The resulting dose volume histograms showed negligible differences between 150 and 200 iterations for all regions, indicating that the optimization had effectively converged by this point. The convergence results are presented in **Supplementary Figure 1**.

Second, MF was reduced stepwise by 0.2 from 5.0. At each MF level, the plan was re-optimized for 200 iterations with the same optimization objectives and dose constraints, and high-resolution dose calculation was performed. The step size of 0.2 was selected as a compromise between threshold resolution and planning workload.

Third, each reduced-MF plan was independently reviewed by two radiation oncologists using a structured evaluation checklist (**Table 2**). Plan acceptability was determined based on three mandatory domains: (1) target coverage requirements (e.g., V_95%_≥95% and absence of cold spots within the PTV); (2) hotspot control, ensuring that D_max_ remained within acceptable limits and that no hotspots were located within serial organs; and (3) organ at risk (OAR) sparing in accordance with site-specific institutional dose constraint schedules (**Supplementary Table 1**). Each reviewer performed the assessment independently. A plan was classified as clinically acceptable only if all mandatory criteria were satisfied. Discrepancies between the two reviewers were resolved through consensus discussion.

**Table 2:** Structured criteria used for plan acceptability evaluation.

Evaluation domain	Objective criteria	Qualitative assessment	Mandatory criterion
Target coverage	V_95%_≥95% of the prescribed dose	No cold spots within the PTV; dose distribution consistent with geometric boundaries.	Yes
Hotspot control	D_max_ ≤ 110% of the prescription dose	No hotspots located inside serial organs-at-risk (e.g., spinal cord, brainstem).	Yes
OAR sparing	According to site-specific institutional dose-constraint tables (**Supplementary Table 1**)	No unexpected high-dose regions; dosimetric pattern anatomically reasonable.	Yes

Notes: 1. Mandatory criterion indicates that failure of any listed item results in the plan being classified as clinically unacceptable. 2. OAR dose limits follow institutional standards and vary by anatomical site; full constraints are provided in **Supplementary Table 1**. 3. Two radiation oncologists independently evaluated each plan using this checklist; disagreements were resolved by consensus.

Because MF was reduced in discrete steps, the gantry period did not necessarily reach exactly the mechanical lower limit of 11.8 s at a clinically useful MF level. Therefore, for efficiency-limited cases, MF_limit_ was operationally defined as the last clinically acceptable MF before the step-down sequence entered the mechanical gantry-period plateau. Plateau entry was identified when further MF reduction resulted in a gantry period at the mechanical lower limit of approximately 11.8 s without a meaningful reduction in delivery time.

If plan quality became clinically unacceptable before plateau entry, the last clinically acceptable MF was defined as MF_limit_. Under this condition, MF_limit_ and MF_min_ were identical by definition. This condition was termed the clinical quality-limited pathway.

For efficiency-limited cases, MF reduction was continued for research purposes after MF_limit_ to identify the lowest clinically acceptable MF, denoted MF_min_. The plans generated at MF_limit_ and MF_min_ were labeled P-MF_limit_ and P-MF_min_, respectively. P-MF_5_ and P-MF_min_ were not intended as routine clinical planning choices; they were included to characterize the relationship between MF, gantry period, LOT distribution, and plan quality.

### 2.3 Gantry period and leaf-open-time analysis

For each MF level in the step-down sequence, the theoretical gantry period reported by the treatment planning system was recorded. The relationship between MF and gantry period was analyzed to determine whether MF reduction produced a continuous decrease in treatment efficiency or reached a mechanical plateau.

LOT characteristics were extracted from the sinogram files using in-house MATLAB scripts. The following parameters were calculated: maximum LOT (LOT_max_), mean LOT (LOT_mean_), the proportion of leaf openings shorter than 100 ms (%LOT < 100 ms) (14), and T_gap_. T_gap_ was defined as the time interval between the available projection time and LOT_max_ (15). These parameters were compared among P-MF_5_, P-MF_limit_, and P-MF_min_ to assess how MF reduction affected fluence modulation and delivery timing.

### 2.4 Dosimetric evaluation and patient-specific QA

To evaluate the clinical feasibility of the proposed strategy, delivery time, dosimetric indices, and pretreatment verification results were compared between P-MF_limit_ and P-MF_min_.

Target dose conformity and homogeneity were assessed using the conformity index (CI) (16) and homogeneity index (HI) (17). CI and HI were calculated as follows:

CI = (V_T,ref_ × V_T,ref_)/(V_T_ × V_ref_)
HI = (D_2%_ − D_98%_)/D_50%_

where V_T,ref_ is the target volume covered by the reference isodose, V_ref_ is the total volume enclosed by the reference isodose, and V_T_ is the target volume. D_2%_, D_50%_, and D_98%_ represent the doses received by 2%, 50%, and 98% of the target volume, respectively. A CI closer to 1 indicated better conformity, and a lower HI indicated better dose homogeneity.

For single-prescription cases, CI and HI were calculated for the planning target volume (PTV). For simultaneous integrated boost cases, the high-dose PTV (PTV_boost_) and elective PTV were assessed using their respective criteria.

For the OAR analysis, all clinically relevant OAR dose metrics defined in the original plan objectives were included as paired observations and pooled across patients and anatomical sites. D_max_-based and D_mean_-based metrics were analyzed separately. Because multiple OAR metrics could be extracted from the same patient and the included OARs differed among anatomical sites, this pooled analysis was considered exploratory and was not intended to provide organ-specific statistical inference. A total of 74 paired D_max_ metrics and 50 paired D_mean_ metrics were included in the pooled OAR analysis.

Pretreatment dose verification was performed using the ArcCHECK detector array with a PMMA CavityPlug. Each plan was recalculated on the ArcCHECK phantom in the DQA workstation and exported via DICOM. Measured absolute dose distributions were compared with calculated distributions using global gamma analysis with a 10% low-dose threshold. Gamma pass rates were evaluated using 3%/2 mm, 2%/2 mm, and 1%/1 mm criteria. A global gamma pass rate of at least 90% under the 3%/2 mm absolute-dose criterion was used as the clinical acceptance threshold (18).

### 2.5 Statistical analysis

Statistical analyses were performed using SPSS version 27.0. Continuous variables were summarized as median and interquartile range because normality could not be assumed. Paired comparisons between P-MF_limit_ and P-MF_min_ were performed using the Wilcoxon signed-rank test. All tests were two-sided, and p < 0.05 was considered statistically significant.

## 3 Results

### 3.1 Gantry period response to MF reduction

All 20 cases followed the efficiency-limited pathway. In each case, the gantry period decreased with MF reduction and subsequently entered the mechanical plateau at approximately 11.8 s. Because MF was reduced in 0.2 increments, the gantry period did not necessarily reach exactly 11.8 s at the selected stopping point. Therefore, MF_limit_ was identified as the last clinically acceptable MF before plateau entry. The quantitative delivery-efficiency metrics are summarized in **Table 3**, and site-specific results are provided in **Supplementary Table 2**.

**Table 3:** Delivery-efficiency metrics for P-MF_5_, P-MF_limit_, and P-MF_min_ in the overall cohort (N = 20).

Metric	P-MF_5_	P-MF_limit_	P-MF_min_
MF	5.0 (5.0, 5.0)	2.3 (2.2, 2.8)	1.8 (1.8, 2.2)
Gantry period (s)	26.9 (22.9, 28.9)	12.6 (12.3, 12.9)	11.8 (11.8, 11.8)
Delivery time (min)	9.6 (7.4, 13.0)	5.3 (4.0, 6.8)	5.0 (3.7, 6.6)

MF, modulation factor.

Values are reported as median (interquartile range).

**Figure 2:**
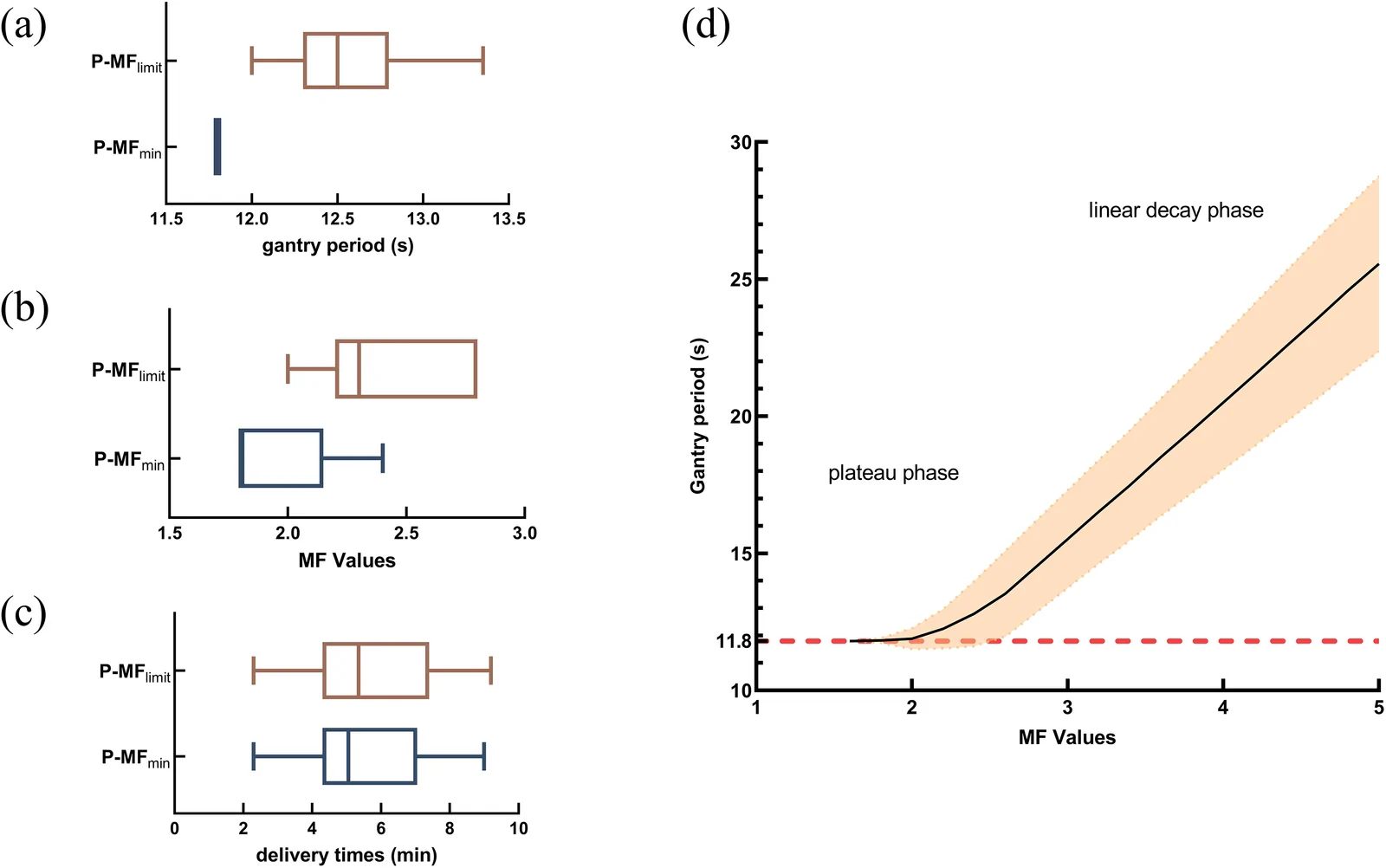
Gantry-period response to MF reduction. **(a)** Distribution of gantry periods for P-MF_limit_ and P-MF_min_. P-MF_limit_ represented the last clinically acceptable plan before gantry-period plateau entry, whereas P-MF_min_ was generally located within the plateau region. **(b)** Distribution of MF_limit_ and MF_min_ values. **(c)** Delivery time comparison between P-MF_limit_ and P-MF_min_. **(d)** Overall relationship between MF and gantry period, showing an initial decreasing phase followed by a mechanical plateau. The dashed red line indicates the mechanical lower limit of 11.8 (s) The shaded band represents interquartile variability.

As shown in **Figures 2a, b**, P-MF_limit_ plans were located immediately before the gantry-period plateau, whereas further MF reduction to MF_min_ led to gantry periods clustered at the mechanical lower limit of approximately 11.8 s. Delivery times were comparable between P-MF_limit_ and P-MF_min_ (**Figure 2c**), indicating that reducing MF beyond MF_limit_ provided no meaningful delivery-efficiency gain.

The overall MF gantry-period relationship showed a biphasic pattern (**Figure 2d**): an initial decreasing phase followed by a mechanical plateau. In this cohort, the pre-plateau MF_limit_ values ranged from 2.0 to 2.8, depending on the patient. MF_min_ values were lower than or equal to MF_limit_ values and were generally located within the plateau region.

### 3.2 Leaf-open-time characteristics

**Figure 3:**
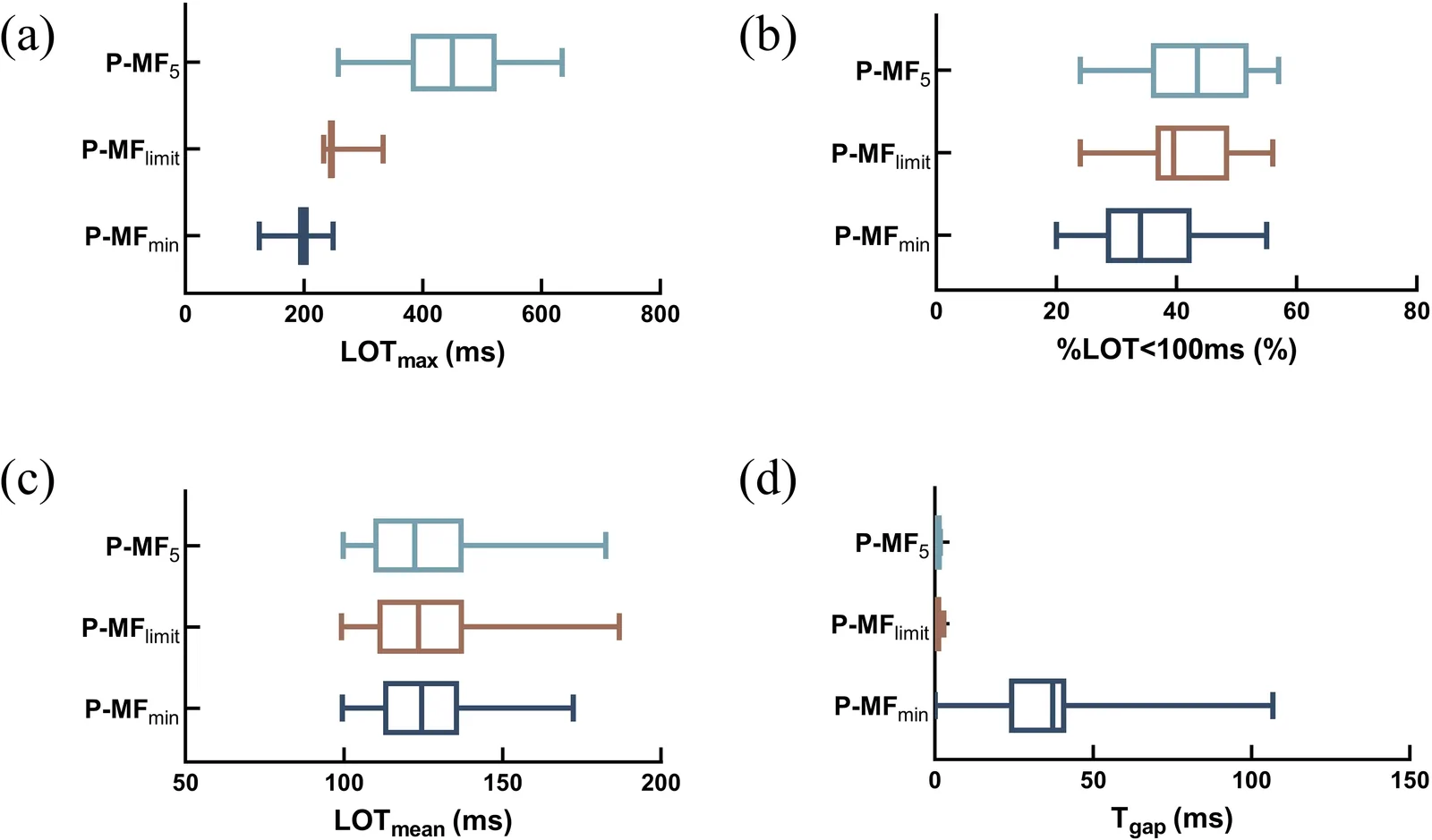
Effects of MF reduction on leaf-open-time characteristics. **(a)** LOT_max_ decreased progressively from P-MF_5_ to P-MF_limit_ and P-MF_min_. **(b)** The percentage of very short leaf openings (%LOT < 100 ms) decreased with MF reduction. **(c)** LOT_mean_ showed limited variation across MF levels. **(d)** T_gap_ increased markedly in P-MF_min_, indicating idle time within the fixed projection window after gantry-period plateau entry.

LOT characteristics changed systematically with MF reduction (**Figure 3**). LOT_max_ decreased from P-MF_5_ to P-MF_limit_ and further to P-MF_min_, reflecting the reduced upper bound of leaf opening as MF decreased. LOT_mean_ showed only limited variation across the three MF levels, suggesting that the average delivery fluence was relatively preserved despite reduced modulation range. LOT-related quantitative metrics for the three MF levels are summarized in **Table 4**.

**Table 4:** Leaf-open-time characteristics for P-MF_5_, P-MF_limit_, and P-MF_min_ in the overall cohort (N = 20).

Metric	P-MF_5_	P-MF_limit_	P-MF_min_
LOT_max_ (ms)	449.9 (379.7, 524.2)	247.4 (240.1, 250.7)	194.2 (190.6, 208.0)
LOT_mean_ (ms)	122.3 (109.3, 137.8)	123.6 (110.6,137.9)	124.5 (112.4, 136.3)
%LOT<100 ms %	43.5 (35.8, 52.0)	39.5 (36.5, 48.8)	34 (28.3, 42.5)
T_gap_ (ms)	1.0 (0.6, 1.4)	0.8 (0.4, 1.6)	37.3 (23.4, 41.6)

LOT, Leaf-open-time; T_gap_, the time interval between the available projection time and LOT_max_.

Values are reported as median (interquartile range).

The proportion of very short leaf openings (%LOT < 100 ms) also decreased with MF reduction. This indicates that lowering MF compressed the LOT distribution and reduced both very long and very short opening events.

A distinct change was observed for T_gap_. T_gap_ remained close to zero for P-MF_5_ and P-MF_limit_, indicating that LOT_max_ nearly occupied the available projection time before plateau entry. In contrast, T_gap_ increased markedly in P-MF_min_. This suggests that after the gantry period had entered the mechanical plateau, further MF reduction shortened LOT_max_ without shortening the projection time, thereby creating idle time within the projection window.

### 3.3 Dosimetric comparison and gamma verification

Both P-MF_limit_ and P-MF_min_ met the predefined clinical acceptability criteria. However, P-MF_min_ showed a trend toward less favorable dosimetric performance (**Table 5**). Compared with P-MF_min_, P-MF_limit_ achieved slightly better PTV_boost_ homogeneity and conformity. The differences in PTV_boost_ HI and PTV_boost_ CI were statistically significant, whereas PTV CI did not differ significantly between the two plans.

**Table 5:** Comparison of target dosimetric metrics, exploratory pooled OAR metrics, and gamma pass rates between P-MF_limit_ and P-MF_min_.

Variable	P−MF_limit_	P−MF_min_	Z value	P value
PTV_boost_ HI	0.04 (0.03–0.07)	0.05 (0.03–0.08)	-2.59	0.010
PTV_boost_ CI	0.79 (0.70–0.91)	0.77 (0.69–0.88)	-2.50	0.012
PTV CI	0.82 (0.77–0.89)	0.81 (0.74–0.88)	-1.66	0.096
OAR D_max_ (Gy)	43.5 (26.3–56.5)	44.1 (27.9–59.2)	-4.70	<0.001
OAR D_mean_ (Gy)	27.3 (18.3–36.6)	27.5 (20.0–37.5)	-6.00	<0.001
Gamma 3%/2mm (%)	99.5 (98.2–99.7)	99.1 (98.4–99.7)	-0.07	0.943
Gamma 2%/2mm (%)	97.6 (95.0–98.8)	95.7 (93.7–98.5)	-0.85	0.394
Gamma 1%/1mm (%)	85.3 (79.5–90.5)	79.4 (75.9–85.1)	-2.46	0.014

OAR D_max_ and D_mean_ were analyzed as exploratory pooled metrics across all clinically relevant OAR objectives (74 paired D_max_ metrics and 50 paired D_mean_ metrics). Multiple OAR metrics could be extracted from the same patient; therefore, the OAR p values should be interpreted descriptively rather than as organ-specific statistical inference.

Values are reported as median (interquartile range).

In the exploratory pooled OAR analysis, D_max_ and D_mean_ were higher in P-MF_min_ than in P-MF_limit_. These pooled findings indicate an overall trend toward less favorable OAR sparing after MF reduction beyond MF_limit_, but they should not be interpreted as organ-specific effects. Gamma pass rates were high for both plans under the 3%/2 mm and 2%/2 mm criteria, with no significant differences. Under the stricter 1%/1 mm criterion, P-MF_limit_ achieved a significantly higher pass rate than P-MF_min_. These results indicate that MF reduction below MF_limit_ did not improve efficiency and may modestly compromise dosimetric quality and delivery robustness.

### 3.4 Comparison with clinically selected MF values

**Figure 4:**
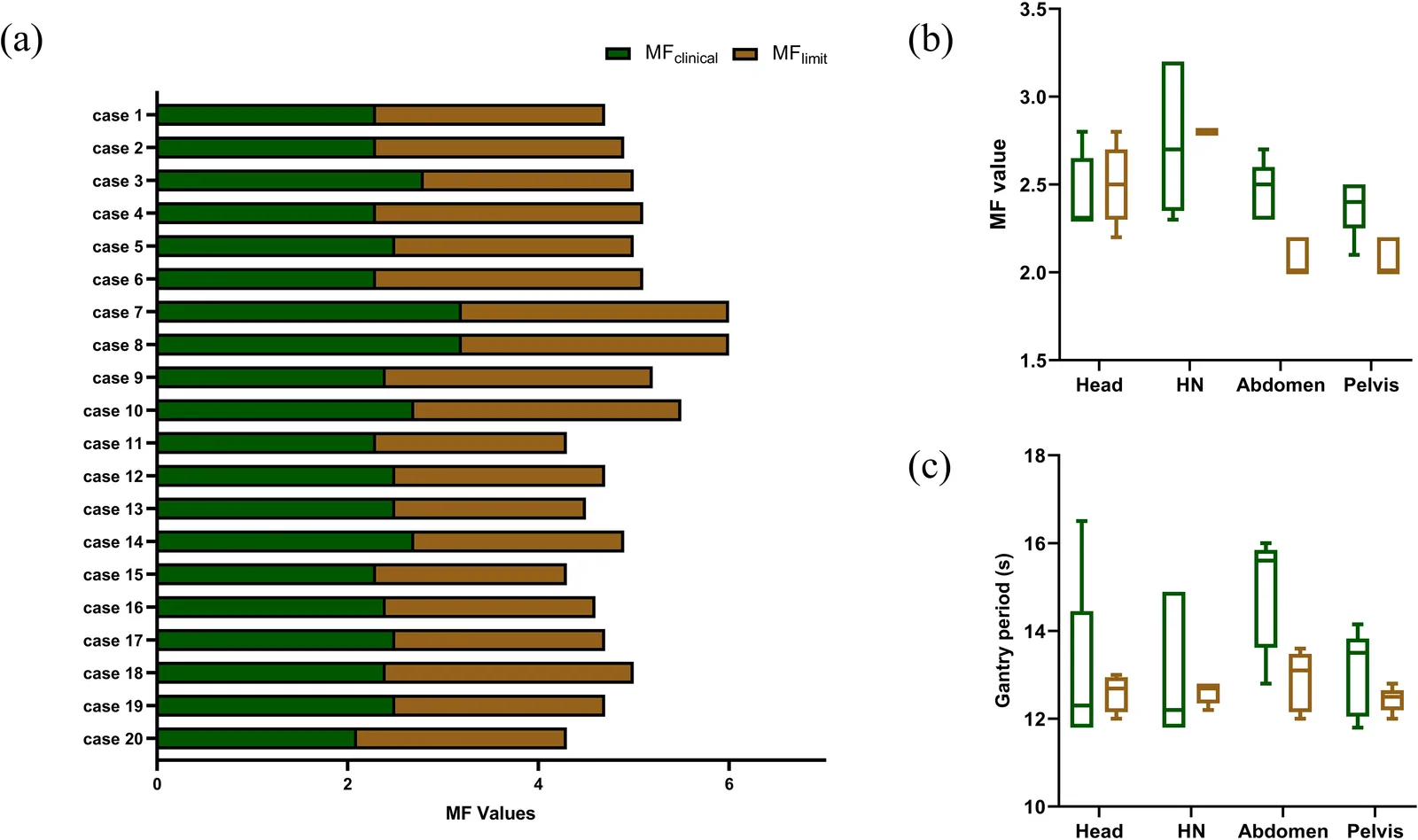
Comparison between clinically selected MF values and gantry-period-guided MF_limit_ values. **(a)** Case-by-case comparison of MF_clinical_ and MF_limit_. **(b)** Distribution of MF_clinical_ and MF_limit_ across anatomical regions. **(c)** Corresponding gantry periods in the original clinical plans and P-MFlimit plans. All clinical and experimental plans used the same field width of 2.5 cm and pitch of 0.287.

**Figure 4** compares the clinically selected MF values with the gantry-period-guided MF_limit_ values. The MF values used in the original clinical plans showed considerable variability among patients, ranging from 2.1 to 3.2. In contrast, the MF_limit_ values obtained through the step-down workflow were distributed within a somewhat narrower range of 2.0 to 2.8.

At the anatomical-site level, clinical MF values also showed greater variability, particularly in head-and-neck and abdominal cases. Although the MF_limit_ values exhibited a narrower distribution within this limited cohort, the small sample size (five cases per site) limits any site-specific interpretation. These observations should therefore be viewed as preliminary, suggesting only that the gantry-period-guided workflow may help avoid unnecessarily high MF settings in some cases.

## 4 Discussion

This study provides three main findings. First, MF reduction showed a biphasic relationship with gantry period, with a decreasing phase followed by a mechanical plateau. Second, LOT analysis demonstrated that further MF reduction beyond the pre-plateau MF_limit_ increased idle time rather than improving delivery efficiency. Third, in this limited cohort, the MF_limit_ plans remained clinically acceptable and avoided the unnecessary loss of modulation seen at MF_min_. These observations suggest that the MF level immediately before plateau entry may serve as a practical reference point for MF reduction, although the evidence remains preliminary given the pilot nature of the study.

The gantry-period plateau provides the physical basis for the proposed strategy, but the practical stopping point is defined immediately before plateau entry. In the decreasing phase, lowering MF reduces the allowed maximum LOT and therefore reduces the time required for each projection, leading to a shorter gantry period. However, when further MF reduction causes the gantry period to enter the mechanical lower limit of approximately 11.8 s, the delivery system cannot further accelerate. Below this threshold, MF reduction continues to alter the LOT distribution but provides little or no delivery-time benefit. This explains why P-MF_min_ did not reduce delivery time compared with P-MF_limit_.

LOT analysis further clarified this mechanism. LOT_max_ decreased progressively as MF was reduced, while LOT_mean_ remained relatively stable. This suggests that the LOT distribution became compressed around its mean. At MF_limit_, T_gap_ remained close to zero, indicating efficient use of the projection window. In contrast, T_gap_ increased markedly below MF_limit_, meaning that leaves closed earlier while the projection window remained fixed by the mechanical gantry-period limit. This idle time represents unused delivery capacity rather than improved efficiency. Consequently, reducing MF below MF_limit_ may remove useful modulation without providing a time benefit. Although these patterns align with the mechanical constraints of HT, the numerical thresholds observed here were obtained using a single combination of field width and pitch and should not be generalized without further validation.

From a clinical planning perspective, the proposed strategy may offer a simple and reproducible rule for MF selection. Previous studies have explored several MF optimization strategies (7–13). Boyd et al. (11) introduced a sequence of decreasing MF values based on the first two moments (mean and standard deviation) of the LOT distribution, using the central limit theorem. Shimizu et al. (12) suggested using the mean actual modulation factor (MF_actual_) from previously treated cases as the initial MF (MF_initial_), followed by stepwise increases up to an upper limit (MF_UL_) derived from the mean and standard deviation of MF_actual_ when further dosimetric improvement was needed. De Kerf et al. (13) generated multiple plans for each patient using different combinations of pitch and MF, and then evaluated the trade-off between treatment time and plan quality through Pareto frontier analysis. Although these methods are valuable, they may require additional planning workload or may not be easily applied to every patient. In contrast, the present strategy uses a parameter already reported by the treatment planning system—the gantry period—to identify the onset of the efficiency boundary. Within this limited cohort, this appeared to reduce some of the empirical variability in MF settings, but larger and more diverse datasets would be needed to determine whether such an effect is generalizable.

Compared with P-MF_min_, P-MF_limit_ maintained clinically acceptable target coverage, OAR sparing, and pretreatment verification performance. The absence of meaningful delivery-time reduction below MF_limit_ indicates that further MF reduction is counterproductive. Although both plans remained clinically acceptable in this cohort, the stricter 1%/1 mm gamma results and the dosimetric trends suggest that excessive MF reduction may reduce plan robustness. Because the OAR findings were derived from pooled metrics across different organs and anatomical sites, they should be interpreted as overall trends rather than organ-specific effects. Taken together, these observations suggest that MF_limit_ may offer a more balanced compromise between delivery efficiency and plan quality than MF_min_, although larger studies are needed to confirm this.

An important feature of the proposed workflow is the first-limiting-condition principle. In this study, all cases happened to enter the efficiency-limited pathway before any loss of clinical acceptability. In principle, the same workflow could be applied to cases in which plan quality fails before plateau entry. In such cases, the last clinically acceptable MF would define MF_limit_ as a clinical-quality boundary rather than a pre-plateau efficiency boundary. However, this interpretation should be viewed cautiously, as the present results are based on a pilot cohort.

This study has several limitations. First, all plans were generated using a fixed FW of 2.5 cm and a fixed pitch of 0.287. Therefore, the numerical MF_limit_ values observed in this study should not be regarded as universal. Different FW and pitch combinations may shift the gantry-period plateau and should be evaluated separately. Second, the MF step size of 0.2 may introduce discretization uncertainty in identifying MF_limit_. Future studies may assess whether smaller MF intervals improve threshold precision. Third, the sample size was limited to 20 cases across four anatomical regions and should be considered pilot in nature. With only five cases per site, the findings are informative for exploring the methodology but are insufficient to support broad conclusions regarding generalizability across disease sites. Larger, multi-institutional datasets will be needed to validate the robustness and applicability of the workflow. In addition, OAR D_max_ and D_mean_ were analyzed using exploratory pooled metrics because the cohort included multiple anatomical sites and only five cases per site. The pooled OAR results should be interpreted as indicating an overall trend rather than organ-specific statistical effects. Finally, all evaluations were performed retrospectively using in-silico planning and pretreatment QA. Prospective workflow implementation would be required to assess its impact of this approach on clinical workflow and planning efficiency.

## 5 Conclusions

Under fixed field width and pitch, the onset of the gantry-period plateau provided a practical physical indicator of when further MF reduction was unlikely to improve delivery efficiency. Within this pilot cohort, the proposed MF_limit_ strategy identified the last clinically acceptable MF before further reduction becomes inefficient or clinically unfavorable. These preliminary findings suggest that gantry-period-guided MF selection may provide a simple and clinically implementable approach to inform MF choice and help avoid unnecessarily low modulation settings. However, the numerical thresholds observed here are configuration-specific and should not be generalized without further validation.

## Data Availability

The original contributions presented in the study are included in the article/**Supplementary Material**. Further inquiries can be directed to the corresponding author.

## Supplementary material

Supplementary Figure 1 Optimization convergence across anatomical regions. DVH comparison for plans optimized with 25, 50, 150, and 200 iterations for four representative cases.

Supplementary Table 1 Site-specific OAR dose constraint summary. Notes: OARs such as oral mucosa and laryngeal substructures follow institutional guidelines due to anatomic variability; these constraints were applied consistently across plans.

Supplementary Table 2 Site-specific delivery-efficiency metrics for P-MF_5_, P-MF_limit_, and P-MF_min_. Values are reported as median (interquartile range). MF: modulation factor.
