# Supplementary material for: Gantry-period-guided modulation factor selection in helical tomotherapy: effects on delivery efficiency, dosimetry and verification accuracy
Source: Front Oncol. 2026 Aug 14;16:1891272. doi: 10.3389/fonc.2026.1891272 (PMC13521858; doi:10.3389/fonc.2026.1891272)
Supplement: Supplementary file 1 [file Supplementaryfile1.docx]

**Table S1. Site-specific OAR dose constraint summary**

| **Anatomical site** | **Organ-at-risk** | **Dose constraints (institutional standards)** |
| --- | --- | --- |
| **Head** | Spinal cord | D_max_ < 45 Gy |
|  | Brainstem | D_max_ < 54 Gy(preferred)  D_max_≤60Gy（acceptable） |
|  | Optic nerves (L/R) | D_max_ < 54 Gy(preferred)  D_max_≤60Gy（acceptable） |
|  | Optic chiasm | D_max_ < 54 Gy(preferred)  D_max_≤60Gy（acceptable） |
|  | Lens (L/R) | D_max_ < 9 Gy |
|  | Inner ear | D_mean_ < 50 Gy; D_max_ ≤ 60 Gy |
| **Head & Neck** | Parotid gland | D_mean_ < 26 Gy; V_30_ < 50% |
| **Abdomen** | Liver | D_mean_ ≤ 30 Gy; V_40_ < 20% |
|  | Stomach | V_40_ ≤ 40% |
|  | Duodenum | D_max_ < 52 Gy |
|  | Kidneys (bilateral) | D_mean_ ≤ 18 Gy; V_20_ < 30% |
|  | Small intestine | D_max_＜54Gy，V_50_≤2% |
| **Pelvis** | Rectum | V_50_ < 50% |
|  | Bladder | V_50_ < 50% |
|  | Femoral heads (L/R) | V_50_ < 5% |
|  | Pelvic bone marrow | V_30_ < 50% |
|  | Ovaries | D_max_ < 12 Gy |
|  | Testes | D_max_ < 4 Gy |

Notes: OARs such as oral mucosa and laryngeal substructures follow institutional guidelines due to anatomic variability; these constraints were applied consistently across plans.

**Table S2. Site-specific delivery-efficiency metrics for P-MF_5_, P-MF_limit_, and P-MF_min_. Values are reported as median (interquartile range).**

| **Anatomical site** | **Metric** | **P-MF_5_** | **P-MF_limit_** | **P-MF_min_** |
| --- | --- | --- | --- | --- |
| **Head (n=5)** | **MF** | 5.0 (5.0, 5.0) | 2.5 (2.3, 2.7) | 2.0 (1.8, 2.2) |
|  | **Gantry period (s)** | 24.8 (22.8, 28.5) | 12.7 (12.2, 12.9) | 11.8 (11.8, 11.8) |
|  | **Delivery time (min)** | 6.6 (4.1, 9.9) | 3.7 (2.9, 4.6) | 3.6 (2.8, 4.2) |
| **Head & Neck (n=5)** | **MF** | 5.0 (5.0, 5.0) | 2.8 (2.8, 2.8) | 2.2 (2.1, 2.2) |
|  | **Gantry period (s)** | 22.7 (22.3, 23.0) | 12.7 (12.4, 12.8) | 11.8 (11.8, 11.8) |
|  | **Delivery time (min)** | 9.5 (8.4, 12.1) | 5.4 (5.2, 7.1) | 5.1 (5.0, 6.7) |
| Abdomen (n=5) | **MF** | 5.0 (5.0, 5.0) | 2.0 (2.0, 2.2) | 1.8 (1.8, 1.8) |
|  | **Gantry period (s)** | 32 (28.1, 35.7) | 13.1 (12.2, 13.5) | 11.8 (11.8, 11.8) |
|  | **Delivery time (min)** | 8.3 (7.3, 13) | 4.6 (4.1, 5.3) | 4.3 (3.8, 4.9) |
| Pelvis (n=5) | **MF** | 5.0 (5.0, 5.0) | 2.2 (2.2, 2.4) | 1.8 (1.8, 1.9) |
|  | **Gantry period (s)** | 27.4 (25.1, 28.5) | 12.5 (12.2, 12.7) | 11.8 (11.8, 11.8) |
|  | **Delivery time (min)** | 13.0 (11.3,17.8) | 7.5 (6.5, 8.4) | 7.1 (6.2, 8.2) |

MF: modulation factor

**Supplementary Figure S1. Optimization convergence across anatomical regions.** DVH comparison for plans optimized with 25, 50, 150, and 200 iterations for four representative cases.
